# Understanding rural Chinese residents’ perceptions of palliative care: a grounded theory study of beliefs about a good death

**DOI:** 10.1186/s12904-026-02275-x

**Published:** 2026-08-07

**Authors:** Shenglong Sun, Shengyu Zhao, Shuai Yuan, Yunling Wang

**Affiliations:** 1 School of Political Science and Law, University of Jinan, Jinan, Shandong China; 2 Research Center for Palliative Care and Life-and-Death Education, University of Jinan, Jinan, Shandong China; 3 Centre for Biomedical Ethics, National University of Singapore, Singapore, Singapore

**Keywords:** Palliative care, China, Rural China, Older adults, Good death, Grounded theory, Family caregiving

## Abstract

**Background:**

Palliative care development in China remains marked by urban-rural inequality, with service expansion concentrated mainly in urban and better-resourced settings. Older residents in rural areas may therefore face limited access to professional palliative care, despite substantial care needs. This study aimed to explore rural Chinese residents’ perceptions of, needs for, and underlying decision-making logic regarding palliative care, to inform the development of culturally responsive rural palliative care services.

**Methods:**

This qualitative study used purposive sampling to recruit rural residents from nine provinces and one municipality in the Chinese mainland. After quality screening, 39 interviews involving 43 participants were included and analysed using a grounded theory approach.

**Results:**

The analysis generated the “Rural Belief about Good Death” framework, comprising four interrelated dimensions: familism as the core principle, attachment to ancestral homeland as the affective orientation, traditional filial piety as the ethical norm, and family economic security as the threshold condition. Participants’ attitudes towards palliative care were not shaped by medical considerations alone, but were embedded in family-centred, place-based, morally charged, and economically constrained understandings of a good death.

**Conclusions:**

Rural palliative care in China should be developed through locally responsive models that preserve family involvement, support home-oriented care, translate palliative care into culturally meaningful terms, and reduce financial barriers. The framework offers empirical and theoretical insights for developing palliative care in rural and resource-limited settings.

**Supplementary Information:**

The online version contains supplementary material available at https://doi.org/10.1186/s12904-026-02275-x.

## Introduction

The accessibility of palliative care in rural areas remains a global challenge. Although rural residents account for nearly half of the world’s population, their access to palliative care is often limited by service unavailability, geographic isolation, resource constraints, shortages of specialised teams, underdeveloped professional training, inappropriate payment models, adverse social determinants of health, and low public awareness [1–6]. These challenges highlight the need for effective approaches to delivering palliative care in resource-limited rural settings.

Similar challenges are evident in China, where rural areas face substantial demographic pressures and uneven service development. According to the Seventh National Population Census, the proportions of people aged 60 years and over and 65 years and over in rural China are 23.81% and 17.72%, respectively. These figures are 7.99 and 6.61% points higher than those in urban areas [7], indicating considerable care needs among rural older adults. However, the coverage of palliative care services in rural China remains inadequate. Since 2017, China has launched three batches of national palliative care pilot projects to promote service development; yet across the 185 pilot counties and districts included in these projects, only two are explicitly identified as rural counties, while most pilot sites are located in urban or better-resourced settings [8–11]. This distribution reflects, and may further reinforce, uneven development in which palliative care expansion remains concentrated in urban settings while rural areas continue to lag behind [10, 11].

Beyond these access-related challenges, rural Chinese contexts may also involve culturally specific understandings of death, family responsibility, place, and economic security that influence perceptions of palliative care and expectations of a good death [12, 13]. However, these perspectives remain underexplored in the existing literature, particularly in relation to the development of palliative care services in rural settings. To further investigate this phenomenon, this study adopts a grounded theory approach to explore rural Chinese residents’ perceptions of, and needs for, palliative care. Based on the data, the study develops the framework of “Rural Belief about Good Death” to conceptualise the cultural and practical meanings through which rural residents understand palliative care. This framework may offer theoretical insights into the localisation of palliative care in rural China, while also enriching global academic discussions and informing practice in rural areas with similar resource constraints and cultural backgrounds.

## Methods

### Study design

This study adopted a qualitative grounded theory approach to explore rural Chinese residents’ understandings of, and needs for, palliative care. Grounded theory was considered appropriate because the study aimed not only to describe participants’ perspectives, but also to develop an empirically grounded conceptual framework explaining how rural residents make sense of palliative care, death, dying, and caregiving in their local context. Participants were invited to take part in semi-structured interviews, which enabled flexible exploration of topics related to palliative care and end-of-life issues while allowing them to raise issues that were meaningful to their own lives.

The semi-structured interview guide was informed by previous literature on palliative care and the practical context of palliative care provision in rural China (Supplementary Material 1). This flexible format was particularly suitable for the study because palliative care and death-related topics can be culturally sensitive in Chinese contexts, and such sensitivity may be especially salient in rural communities [11, 14]. It allowed participants to manage the pace of the conversation and to introduce issues that they considered salient to their own experiences.

### Participants and recruitment

Participants were purposively sampled according to the following criteria: (1) aged 40 years or older; (2) held rural household registration; (3) full mental capacity; (4) capable of independent verbal communication; (5) owned a legally registered rural homestead; (6) did not own urban residential property; and (7) had continuously resided in a rural area for at least three years. The final criterion was used to ensure that participants’ accounts were grounded in lived experience of rural communities. Participants’ rural residency was confirmed during the recruitment process.

Recruitment was conducted through two routes. First, the researchers contacted staff members of local village committees, either in person or by telephone, to introduce the study. Staff members who agreed to assist helped identify eligible participants and approached them with a brief overview of the study. Second, researchers approached potential participants through family members or acquaintances living in rural areas, who were able to confirm participants’ rural residency. In both recruitment routes, these individuals only facilitated initial contact, and participation was confirmed directly with potential participants by the researchers.

To capture diverse rural contexts, participants were recruited from nine provinces and one municipality in the Chinese mainland: Shandong, Shanxi, Hebei, Henan, Hubei, Hunan, Guizhou, Sichuan, Fujian, and Beijing. As the study did not aim to conduct cross-regional comparisons, the number of participants recruited from each province or municipality was not predetermined. Recruitment instead aimed to capture diverse rural contexts and generate sufficient depth and breadth of data to address the study aims.

### Data collection

Data were collected between July and September 2024 by a multidisciplinary research team. The team comprised university professors, medical professionals, social workers, and graduate and undergraduate students recruited from universities across China. Team members had experience in qualitative research and/or palliative care, with disciplinary backgrounds including medicine, nursing, sociology, and social work. Before data collection, all team members completed a four-day training programme to ensure a shared baseline understanding of the core concepts of palliative care and the procedures for conducting semi-structured interviews. During the interviews, interviewers used the same interview guide to support consistency across data collection.

Before each interview, participants were informed of the purpose of the study and their right to withdraw. The interviewer also provided a brief introduction to the basic concepts of palliative care, followed by a comprehension check to ensure that participants had an adequate understanding of the concept. Any misunderstandings were clarified before the interview began.

Interviews were conducted in spaces that offered as much privacy as practicable, either in a private room at a village committee office, in an individual hospital ward, or in the participants’ homes. One-to-one interviews were conducted as the primary data collection method. Group interviews were reserved for participants unable to move to a separate interview room for physical reasons. After additional explanation of the potential limits to privacy in a group setting, participants gave explicit consent to take part in group interviews. To reduce the risk of conformity bias, the moderator encouraged participants to express their views independently and remained attentive to dominant opinions. The group interviews followed the same topic guide as the individual interviews to support consistency in the data collected. The research team acknowledged that group interviews may generate different interactional dynamics from individual interviews; therefore, group interview data were reviewed carefully during analysis for potential conformity effects and compared with individual interview data for consistency.

With participants’ consent, interviews were audio-recorded and transcribed within 24 h. Field notes were taken during the interviews to capture non-verbal expressions, such as gestures and facial expressions.

### Data analysis

This study adopted a Straussian grounded theory approach to data analysis [15, 16]. This approach was particularly suited to the study because the research aimed not only to describe rural residents’ views of palliative care, but also to identify relationships among their cultural, relational, moral, and economic considerations and integrate these into an explanatory framework. The structured procedures of open, axial, and selective coding enabled the analysis to move systematically from initial concepts, to relationships among categories, and finally to the development of the “Rural Belief about Good Death” framework. This was especially valuable in an under-researched area where existing theoretical models were insufficient to explain how familism, place attachment, filial piety, and economic constraints interacted in shaping attitudes towards palliative care. Constant comparison was used throughout the analysis to refine categories and examine their relationships across participants’ accounts. Recognising the potential for researcher influence and over-abstraction, the team conducted regular reflexive debriefings and repeatedly compared emerging categories with the original transcripts. These processes helped ensure that the resulting framework remained grounded in participants’ accounts while allowing systematic conceptual development.

All interviews were transcribed verbatim and anonymised, with names and other identifiable information removed and replaced with participant codes. Before formal coding, interview transcripts underwent quality assessment. Interviews shorter than 30 min were re-examined for the depth and relevance of their substantive content in relation to the study aims. Two researchers independently reviewed the transcripts and discussed any disagreements, with a third researcher consulted where discrepancies remained unresolved.

To enhance analytic rigour and reduce individual interpretive bias, coding was conducted collaboratively by multiple researchers, following the three analytical stages of grounded theory: open coding, axial coding, and selective coding.

Open coding was conducted by the first researcher using NVivo 15. Codes were generated inductively from the raw text, and reference points for each code were recorded to document the frequency and variation of participants’ expressions [17]. The second researcher independently reviewed the appropriateness of the initial codes and discussed disputed items with the first researcher to reach consensus. Any unresolved discrepancies were discussed with a third researcher. Following several rounds of discussion, some codes were revised or merged to refine the open coding framework.

Axial coding was then conducted to explore relationships between the initial concepts and to summarise major categories. Through constant comparison and inductive analysis, the open codes were grouped into axial codes according to conceptual similarity. Selective coding was performed to identify and refine the core categories through the systematic analysis of logical relationships among the categories.

Because data collection was conducted concurrently across the nine provinces and one municipality, saturation was assessed during analysis rather than used prospectively to determine the endpoint of recruitment. The research team first analysed the one-to-one interview transcripts. Data saturation was considered to have been reached, when no substantially new codes, categories, or conceptual insights were identified.

As the interviews were conducted in Mandarin, all quotations were initially translated into English by bilingual members of the research team. To enhance accuracy and consistency, the translations were independently reviewed and cross-checked by other researchers. For culturally specific expressions, such as the idiom “falling leaves return to the root”, the translation retained the original imagery while providing an accompanying explanation in the text to clarify its cultural meaning. This process helped preserve the meaning and nuance of participants’ original expressions in the translated quotations.

### Ethics approval

This study was reviewed and approved by the Ethics Committee of the University of Jinan (approval number: UJN-SSS-2024-001). Before data collection, the researchers explained the study purpose and methods to all participants and assured them of confidentiality. All participants were informed that participation was voluntary and that they could withdraw from the interview at any time without penalty. Written or verbal informed consent was obtained from all participants before the interviews. To protect participants’ privacy, participant identities and village names were anonymised.

### Researchers’ reflexivity and positionality

This study was conducted by a multidisciplinary research team with backgrounds in medicine, nursing, social work, and sociology. These disciplinary perspectives informed the study design, data collection, and interpretation of the findings. Team members with clinical backgrounds were more familiar with biomedical and care-related aspects of palliative care, whereas those with social science and social work backgrounds brought greater attention to participants’ social relationships, family contexts, and lived experiences. While this diversity enabled the data to be examined from multiple perspectives, the team recognised that professional training and prior understandings of palliative care could influence the issues explored during interviews and the interpretation of participants’ accounts. Regular reflexive debriefings were therefore conducted throughout fieldwork to discuss emerging impressions, question disciplinary assumptions, and identify possible differences in interpretation. Data analysis was similarly conducted through iterative discussion among multiple researchers, allowing alternative interpretations to be compared and critically examined before the analytical categories and framework were developed.

Reflexive attention was also given to the relationships through which participants were recruited and interviewed. Some participants were recruited with the assistance of village committees with which members of the research team had established working relationships. This raised the possibility that participants might provide socially desirable responses or moderate their views because of perceived relationships with local intermediaries. However, the researchers conducting the interviews did not have prior personal relationships with the participants. During recruitment and data collection, participants were informed of the purpose of the study, the researchers’ role, and their freedom to express both positive and negative views. Interviews used open-ended and non-judgemental questioning, with follow-up probes used to encourage participants to elaborate on their own experiences and perspectives. These measures were intended to reduce undue influence arising from the recruitment pathway and to create an interview environment in which participants could express their views openly.

## Results

A total of 52 interviews were initially conducted and transcribed. Following the review process, 13 interviews were excluded because they provided insufficient substantive material for analysis. Of the remaining 39 interviews, 36 were one-to-one and three were group interviews (involving three, two, and two participants, respectively). Data saturation was achieved following the analysis of the 21st transcript. However, given potential differences in economic conditions, cultural values, and customs across eastern, central, and western China, the research team analysed all 39 transcripts to capture a broader range of perspectives and greater contextual nuances. Subsequent analysis confirmed that no new codes, categories, or conceptual insights arose after the point of saturation.

### Participant demographics

A total of 43 rural residents participated in the study, representing variation in age, gender, and educational background. The median age of participants was 62 years (range 42–82). Of the participants, 27 were female (62.8%) and 16 were male (37.2%). In terms of education, 23 participants (53.5%) had primary schooling or less, whereas 20 (46.5%) had attended junior high school or above (Table 1).

**Table 1:** Participant demographics (*N* = 43)

Variable	*N* (%)
Age (years) median (range)	62 (42–82)
Gender	
Male	16 (37.21)
Female	27 (62.79)
Education level	
Elementary school or below	23 (53.49)
Junior high school	12 (27.91)
High school or above	8 (18.60)

### Coding results and the “rural belief about good death” framework

As described in the Data analysis section, 32 open codes were identified through open coding, comprising 476 reference points (Table 2). These counts were used descriptively to support transparency in the coding process, rather than as the sole basis for determining analytic significance. And the detailed open codes, including representative quotes, are provided in the supplementary material (Supplementary Material 2). These codes were then organised into eight axial categories: “Views on Life and Death”, “Awareness of Quality of Life and Dignity”, “Perceptions of Medical Care”, “Customs for Handling Postmortem Matters”, “Attitudes Toward Palliative Care”, “Influence of Economic Status on Acceptance of Palliative Care”, “Needs for Palliative Care Services”, and “Expectations for Government Support and Promotion Policies”.

**Table 2:** Coding results

Axial Codes	Open Codes	Reference Points
Views on Life and Death (89)	Cherishing life and fearing death	32
	Falling leaves returning to the root	20
	Life and death as natural	17
	Fearlessness towards death	8
	Dying peacefully in old age	5
	One’s fate is not in one’s own hands	4
	Death as liberation	3
Awareness of Quality of Life and Dignity (118)	Family Fulfilment	39
	Physical and mental well-being	35
	Dying without pain	14
	No lack of material resources	12
	Avoiding futile treatment	9
	Longevity	5
	Not being a burden to family and children	4
Perceptions of Medical Care (85)	Patient care relying on the family	55
	Treating to the end to fulfil filial piety	19
	Patient care through neighbourhood mutual assistance	8
	Prioritizing treatment for the young over the elderly	3
Customs for Handling Postmortem Matters (20)	Dying at home	8
	Family members staying by the dying person’s side	4
	Being laid to rest in peace	3
	Purchasing burial items and dressing the deceased in burial clothes	3
	Not disturbing the deceased	2
Attitudes Toward Palliative Care (64)	Positive acceptance of palliative care	47
	Rejection of palliative care	17
Influence of Economic Conditions on Acceptance of Palliative Care (23)	Poverty hindering rural residents from accepting palliative care	13
	Accepting palliative care only if costs are low	10
Needs for Palliative Care Services (40)	Hoping for professional service support	35
	Expecting home-based care	5
Expectations for Government Support and Promotion Policies (37)	Strengthening policy support	17
	Government increasing financial investment	12
	Hoping for life-and-death education and palliative care publicity	8

**Fig. 1:**
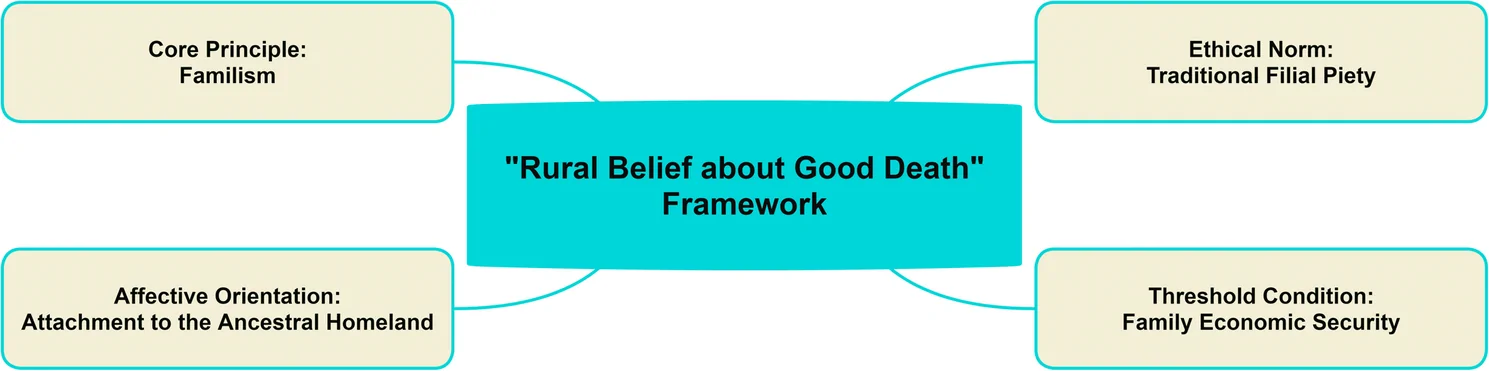
The “rural belief about good death” framework

Analysis of the relationships among the axial categories suggested that participants’ understandings, attitudes, and expectations regarding palliative care were shaped by a broader framework comprising four interrelated dimensions: familism, attachment to ancestral homeland, traditional filial piety, and family economic security as the threshold condition. We termed this framework the “Rural Belief about Good Death” framework (Fig. 1). Through selective coding, the four dimensions were integrated to explain rural Chinese residents’ perceptions of, and needs for, palliative care.

These four categories represent distinct but interconnected dimensions of the framework. Familism identifies the family as the basic unit through which participants understood life, death, caregiving, decision-making, and palliative care. Attachment to ancestral homeland captures the emotional and spatial expectations attached to family-centred dying, especially the importance of home, belonging, and return. Traditional filial piety explains the ethical significance of family participation in palliative care. Family economic security identifies the practical boundary within which care decisions were made.

This framework is used here as an analytic construct developed from the data to explain how participants connected palliative care with broader understandings of a good death in rural life. Together, these categories show that participants’ perceptions of palliative care were not shaped by medical considerations alone, but were embedded in culturally, ethically, and economically situated understandings of life, death, family responsibility, and care. Their reported needs therefore need to be understood in relation to rural customs, family-centred moral expectations, and the practical constraints of economic insecurity and limited service access. Each of these four categories is elaborated in the following subsections, drawing on the empirical evidence.

### Familism as the core principle

Familism formed the core principle of the “Rural Belief about Good Death” framework by positioning the family as the primary unit through which participants understood life, death, caregiving, and palliative care. Participants generally framed palliative care in relation to family responsibilities, family well-being, and the preservation of family integrity.

This family-centred understanding was particularly evident in participants’ accounts of dignity. Dignity was not understood only as an individual condition, but as something realised and preserved within family relationships. Participants suggested that an important condition for maintaining dignity in later life was not being a burden to your children. C17 indicated that “*truth be told*,* not being a burden to your children is the true blessing.*” Avoiding burden was therefore not simply an expression of self-denial. Rather, it allowed older people to continue contributing to the family by reducing pressure on their adult children. As C13 pointed out,

He [the patient] must be feeling really guilty. He’s sick and bedridden, and he thinks he’s just becoming a burden on us. He knows how hard his son works to make money out there.

In this sense, participants’ accounts reflected a form of reverse care, in which older people, despite their vulnerability, continued to care for their family members by limiting the demands placed upon them [18]. The family was also closely associated with quality of life. As C22 stated, “*if the family is a mess*,* life is a mess.*” For participants, well-being in later life was closely connected to family harmony, emotional security, and the stability of everyday family life.

Familism also shaped participants’ expectations of caregiving practice. The familial bond was associated with trust, intimacy, and moral reliability. For this reason, the involvement of non-family caregivers was viewed by some participants with caution. Palliative care professionals could be perceived as “outsiders” whose presence might disrupt the intimacy of family-based care:

C3: “When outsiders come over, no matter how well they speak, they just can’t create that genuine spiritual resonance.”

C8: “If an outsider comes to take care of the elder, we’ll feel uneasy. We think outsiders can’t do this job, because it’s something only their own children should do.”

These accounts suggest that participants did not evaluate palliative care only in terms of clinical effectiveness. They also considered whether care could preserve the relational trust and emotional closeness associated with family caregiving. In this sense, familism provided the standpoint from which participants approached subsequent questions about place of death, filial responsibility, service acceptability, and economic burden. Across the interviews, the family functioned as the default unit of meaning, care, and decision-making. Familism therefore shaped not only participants’ understanding of a good death, but also the conditions under which palliative care could be considered acceptable: care needed to support, rather than replace, the family’s role.

### “Attachment to the ancestral homeland” as the affective orientation

Participants’ attachment to ancestral homeland shaped their sense of belonging and their expectations about where and how a good death should occur. Within the “Rural Belief about Good Death” framework, this attachment gave family-centred dying a spatial, emotional, and ritual orientation. Participants invoked the Chinese idiom “*falling leaves return to the root*” to explain the cultural significance of homecoming at the end of life. A good death was therefore not understood only as the absence of suffering, but also as a form of return: to one’s home, family, local community, and familiar cultural world.

This orientation was most clearly expressed in participants’ preference for dying at home. As C13 stated, “*Within rural beliefs about death*,* it’s not good to die outside. One must die at home.*” Home was described not only as a convenient place of care, but also as a place of safety, reliance, and emotional belonging. C5 explained, “O*lder people prefer being at home more*,* where they feel safe and have someone to rely on.*” These accounts suggest that home carried symbolic significance beyond its practical function. For participants, dying at home helped preserve continuity between the person’s life, family relationships, and local identity.

Attachment to ancestral homeland was also expressed through the expectation of family presence at the end of life. Participants described the presence of children and relatives as an important condition for reducing fear and loneliness at the moment of death. As C8 stated,

When all the children are by their side, it’s quite lively, so the dying elderly won’t feel terrified or lonely. Because life and death are the most important moments in life.

Family companionship was therefore not merely emotional support. It also helped complete the dying person’s life course by locating death within the relational world in which the person had lived.

This orientation further extended into postmortem practices. Open codes such as “not disturbing the deceased” and “purchasing burial items and dressing the deceased in burial clothes” reflected participants’ concern that the deceased should be treated with care, peace, and dignity. These practices suggest that a good death was understood as requiring not only comfort before death, but also ritual completion afterwards. Overall, attachment to ancestral homeland shows that participants’ expectations of a good death were shaped by the desire for spatial return, emotional belonging, and ritual closure. The family was therefore expected to assist the dying person in achieving a good death not only through caregiving, but also through presence, farewell, and culturally meaningful postmortem arrangements.

This attachment also shaped participants’ attitudes towards palliative care, particularly when care was imagined in an inpatient or institution-based form. Hospitalisation could be understood as a separation from the familiar environment in which emotional support, family presence, and ritual closure were expected to occur. Participants’ hesitation towards palliative care often reflected concerns that inpatient care might not provide dying patients with the family presence, emotional support, and cultural familiarity they considered essential to a good death, rather than a rejection of its clinical benefits.

This perspective was reflected in C12’s account:

Oh, I think it’s more convenient at home than in an institution. You know, going to the hospital is just like being in prison; even a healthy person would be worn out after just a few days there.

This finding shows that rural palliative care may be more acceptable when it enables patients to remain close to home, preserves family presence, and accommodates culturally meaningful practices around dying and death.

### “Traditional filial piety” as the ethical norm

If attachment to ancestral homeland shaped the emotional and spatial orientation of a good death, traditional filial piety gave family participation its ethical significance. Participants did not understand family involvement in palliative care only as a practical arrangement. Rather, caring for an older family member was also framed as a moral duty, especially for adult children. As C2 explained, “*Having filial children is a blessing. Follow the elderly’s wishes and don’t upset them – this is happiness.*” In this account, filial piety was associated with emotional support, respect for the older person’s wishes, and the preservation of well-being in later life. In relation to palliative care specifically, some participants expressed openness to the concept, recognising its potential to “*alleviate the pain of loved ones*” (C37) and “*allow older people to pass away peacefully*” (C19).

However, filial piety also created moral pressure around medical decision-making. As discussed in the section on familism, some older participants were reluctant to impose additional caregiving or financial burdens on their families. By contrast, participants speaking from the position of adult children often emphasised the moral and social importance of fulfilling care responsibilities. In these accounts, medical treatment could become closely associated with filial care. Not providing active treatment, even when the expected clinical benefit was limited, could be interpreted as neglecting one’s filial duty and could expose the family to moral criticism. C4 stated, “*Even if the treatment might lead to his death*,* we cannot just let him stay at home untreated.*” C14 similarly explained,

If parents themselves have no hope of recovery, treating them merely prolongs their life by a few days. But if you don’t attempt resuscitation, when this gets out, the neighbour will say ‘this son is the most unfilial’. They fear being called unfilial.

These accounts suggest that the continuation of treatment was not always understood primarily in terms of clinical benefit. It could also function as a visible demonstration of filial responsibility. In this context, the moral assessment of care centred not only on whether treatment improved the patient’s quality of life, but also on whether the family had visibly “done enough”. Patient comfort and quality of life could therefore become secondary to the perceived need to demonstrate filial commitment and avoid social condemnation.

This moral pressure also shaped participants’ attitudes towards palliative care. Hesitation towards palliative care did not necessarily reflect rejection of its clinical aims, but concern that shifting from active treatment to comfort-oriented care might be interpreted as under-fulfilment of filial duty. This concern was reinforced by limited understanding of palliative care and by the tendency to equate filial care with continued medical intervention. During the interviews, some participants recognised the need for clearer public education. As C19 suggested,

I think the government needs to do more to promote education about palliative care, such as playing promotional videos in the villages and holding awareness sessions like they did before, so that everyone can understand what palliative care is all about.

Some participants recognised palliative care as a way to reduce suffering, preserve dignity, and support a peaceful death. These accounts suggest that palliative care could be understood as compatible with, rather than contrary to, filial responsibility.

### “Family economic security” as the threshold condition

While the previous subsections have shown how participants’ views were shaped by family-centred, place-based, and ethical expectations, this final category identifies the practical boundary within which these expectations were negotiated. For participants, decisions about palliative care were financially sensitive. In addition to concerns about social judgement and filial responsibility, expected costs were an important reason for hesitation towards palliative care. As C33 stated, “*rural folks have no money in hand. The problem is having no money.* … *Long-term treatment and care costs are difficult for many rural families to bear.*” This suggests that reluctance to use palliative care did not necessarily reflect a lack of need or a simple preference against such care. Rather, it could reflect a compromise made under conditions of limited household resources.

Participants’ comparisons between palliative care and conventional treatment further illustrated this financial sensitivity. C32 explicitly distinguished between acceptable and unaffordable levels of expenditure: “*A small amount of money is acceptable*,* but none of us are willing or able to afford a large sum*,* right?*” Similarly, C15 assessed the acceptability of palliative care in relation to its comparative cost: “*As long as palliative care costs less than conventional treatment*,* I think it should be acceptable.*”

This account complicates the relationship between filial piety and medical treatment discussed in the previous section. Although continued treatment could be understood as a visible expression of filial responsibility, participants also weighed care options against their affordability and perceived cost-effectiveness. Acceptance of palliative care therefore depended not only on its moral or cultural acceptability, but also on whether it could be reconciled with the family’s economic capacity. When moral obligations, care ideals, and financial limits came into tension, family economic security functioned as the threshold condition that shaped what forms of care were considered practically possible.

This finding shows that the accessibility of palliative care in rural China cannot be understood as a matter of individual preference alone. Participants described how limited household financial resources constrained their ability to access and sustain care. For this reason, participants expected government support to play an important role in reducing financial barriers and expanding rural palliative care provision. As C4 stated, *“In my view*,* if rural areas want to have such palliative care conditions*,* the government must invest real funds in it.*” Public funding and supportive policies were therefore viewed as important conditions for making palliative care more accessible to rural families.

Taken together, these four categories show how the “Rural Belief about Good Death” framework shaped participants’ attitudes towards palliative care. Participants’ acceptance of palliative care was conditional on whether care could preserve family involvement, remain close to home, align with filial responsibility, and avoid imposing unsustainable financial burdens on the household. Their service expectations therefore centred on family-supported, home-oriented, culturally acceptable, and economically accessible care. These findings suggest that rural residents’ needs for palliative care were not separate from their understandings of a good death, but were embedded within the same family-centred, place-based, morally charged, and economically constrained framework.

## Discussion

This study developed the “Rural Belief about Good Death” framework to explain how rural Chinese residents understand and evaluate palliative care. The findings show that attitudes towards palliative care were shaped not by medical considerations alone, but by interconnected relational, place-based, moral, and economic concerns. The Discussion first considers what this framework adds to existing knowledge, then examines how it explains participants’ conditional acceptance of or hesitation towards palliative care, and finally considers the implications for rural palliative care service development.

### Beyond family caregiving: rethinking rural palliative care

This study contributes to existing knowledge of rural palliative care by showing that the family should not be understood only as a source of informal caregiving or practical support. Within the “Rural Belief about Good Death” framework, the family functioned as the broader context through which participants understood care, belonging, moral responsibility, place, and economic decision-making. Home acquired meaning through its association with family presence and continuity, filial responsibility shaped judgments about appropriate care, and financial decisions were made in relation to the welfare of the household as a whole. The framework therefore extends conventional understandings of family-centred palliative care by conceptualising the family not simply as a provider of care, but as the relational, affective, moral, and economic context within which palliative care becomes meaningful.

A second contribution of the framework is that it brings cultural meanings and material constraints into the same explanatory account. Understandings of a good death were shaped not only by values such as familism, attachment to ancestral homeland, and filial responsibility, but also by the practical limits imposed by household economic security. These dimensions were closely interconnected rather than operating as independent influences. Economic constraint was not merely an external barrier encountered after care preferences had been formed; it also shaped judgments about what forms of care were appropriate, worthwhile, and practically possible. The framework therefore suggests that rural end-of-life preferences are produced through the interaction of relational expectations, cultural meanings, moral obligations, and material conditions.

At the same time, the framework should not be interpreted as a fixed or homogeneous account of rural Chinese culture. Participants’ accounts contained tensions and competing orientations, including both fear and acceptance of death, support for continued treatment and concern about unnecessary suffering, and both openness and hesitation towards palliative care. These tensions suggest that the four dimensions of the framework should be understood as organising influences rather than deterministic cultural rules. The framework therefore identifies a situated value logic through which participants interpreted palliative care, while recognising that these values remain open to negotiation, reinterpretation, and change.

The study also contributes to international palliative care literature by offering an analytic framework for examining how locally grounded understandings of a good death shape the acceptability of palliative care in rural and resource-limited settings. The specific meanings attached to family, place, moral responsibility, and economic security will differ across cultural contexts, and the “Rural Belief about Good Death” framework should not be treated as universally applicable in its substantive form. Its broader theoretical relevance instead lies in identifying a set of interconnected questions: who constitutes the relevant unit of care, how place and belonging shape end-of-life preferences, which moral obligations influence judgments about appropriate care, and how material constraints define the boundaries of feasible choices. These questions may provide a useful basis for comparative research and for the development of contextually responsive palliative care models beyond rural China.

### Conditional acceptance: when palliative care fits a good death

The explanatory value of the “Rural Belief about Good Death” framework lies in showing that participants’ attitudes towards palliative care were conditional rather than simply positive or negative. Palliative care became more acceptable when it could preserve family involvement, maintain continuity with home and local belonging, be interpreted as compatible with filial responsibility, and remain economically sustainable for the household. Conversely, hesitation arose when care was perceived as disrupting these relational, spatial, moral, or economic conditions. The framework therefore explains acceptance through four interconnected conditions: relational compatibility, place-based continuity, moral legitimacy, and economic feasibility.

At the relational and place-based level, acceptance depended partly on whether palliative care could preserve family presence and continuity with the dying person’s familiar social and cultural environment. Participants’ hesitation was therefore not necessarily directed towards the clinical value of palliative care itself, but towards forms of care that might separate dying from family involvement, home, and local belonging. From this perspective, the acceptability of palliative care depended partly on whether it could be incorporated into, rather than displace, the relational and spatial conditions that participants associated with a good death.

Moral legitimacy constituted a second condition of acceptance. The tension between alleviating suffering and continuing medical treatment reflected different understandings of filial responsibility. While continued treatment was often perceived as a visible way of fulfilling filial duty, some participants also recognised that palliative care could reduce suffering and support a peaceful death, which they regarded as an important component of a good death. These accounts suggest that filial piety is open to reinterpretation rather than fixed in a single form of practice. When palliative care is understood as compatible with filial responsibility, it can acquire greater moral legitimacy within the local cultural context. An important task for the future development of palliative care in rural China, and potentially in other cultural contexts where family obligations strongly shape palliative care, is therefore to make such compatibility more explicit and culturally intelligible.

Economic feasibility constituted a further boundary of acceptance. Despite its potential clinical benefits, palliative care could be perceived as financially burdensome if it introduced additional costs without offering benefits that participants considered sufficient to justify them. In such circumstances, participants tended to prioritise household economic security and the welfare of the family as a whole. This concern can be understood in relation to financial toxicity, which has been associated with poorer quality of life for patients and their households [19–22]. Participants’ sensitivity to cost should therefore not be interpreted simply as rejection of palliative care, but as a rational compromise arising from the need to balance care needs against limited household resources.

Taken together, these findings show that the family occupies a central but ambivalent position in rural palliative care. It provides the primary context of care, emotional belonging, moral responsibility, and economic decision-making, while also bearing the accumulated pressures associated with caregiving, cultural expectations, filial obligations, and financial constraint. The family should therefore be understood not simply as a cultural resource, but as both the principal context within which palliative care becomes meaningful and a potential site of substantial moral, emotional, and economic burden. This dual position is important for understanding both the conditional acceptance of palliative care and the forms of external support required for its sustainable development.

### From framework to service model: building locally responsive rural palliative care

The “Rural Belief about Good Death” framework suggests that rural palliative care should be developed through service models that are compatible with local expectations of family involvement, place, filial responsibility, and economic security. Rather than simply replicating urban or institution-based models, service development should combine home-oriented care, community support, accessible professional services, culturally grounded public education, and financial protection.

First, home-based palliative care may be particularly acceptable in rural China because it can accommodate residents’ preferences regarding place of death and their cultural, social, and spatial expectations about where to bid farewell [23]. Care at home may also preserve everyday interaction between patients and family members, allowing relatives to remain closely involved in care and to fulfil perceived filial responsibilities. Such involvement may help sustain trust and reduce concerns about the displacement of family roles by unfamiliar professionals. These findings are consistent with international literature showing that home is widely preferred as a place of care and death and that family members often remain a primary source of support in palliative care [24–30]. However, the cultural importance of family involvement should not be interpreted as a reason to substitute family care for professional support. Participants also expressed a clear expectation for professional palliative care, provided that services were accessible and affordable.

A locally responsive model should therefore combine family involvement with broader community and professional support. Existing rural social networks may provide an important source of supplementary informal care. In rural China, kinship ties and long-standing relationships among neighbours and acquaintances can create forms of quasi-familial trust and mutual obligation. Such networks may be particularly important in communities affected by rural-to-urban migration, where the availability of immediate family caregivers is reduced. Existing social relationships could therefore be mobilised as community resources through neighbourhood-based mutual support, complementing rather than replacing family and professional care [31].

At the same time, community support cannot compensate for the absence of formal palliative care infrastructure. Consistent with participants’ expectations, sustained public investment and policy support are needed to develop professional training, service delivery systems, and insurance or subsidy mechanisms. The combined pressures of economic constraint and limited service capacity suggest that rural palliative care should prioritise affordability, accessibility, and adaptability. This approach is consistent with the concept of “inclusive palliative care”, which emphasises low-cost, broad-coverage, and high-quality service provision while also addressing equity and universal access [32, 33]. Government support is therefore essential for reducing out-of-pocket expenditure, strengthening rural service capacity, and making professional palliative care practically accessible to rural families.

Alongside service development, public education should be undertaken in partnership with local communities. Greater understanding of palliative care may help address the perception that comfort-oriented care represents abandonment of treatment. Presenting palliative care as a way to relieve suffering, improve quality of life, respect older people’s wishes, and support a peaceful death may make its goals more intelligible within existing understandings of filial responsibility and a good death [34]. Improved understanding may also facilitate early integration of palliative care into care planning. Earlier integration has been associated with reduced hospitalisation costs and lower financial burden among patients at the end of life [35–38]. Public education should therefore address both the moral concerns surrounding filial responsibility and the practical concerns associated with cost, while translating professional concepts into forms that are meaningful within local cultural contexts.

Beyond rural China, these findings have wider relevance for the development of palliative care in rural and resource-limited settings. Although the specific meanings attached to family, place, moral responsibility, and economic security will differ across contexts, service models elsewhere may face similar challenges in balancing local care practices with professional support, cultural legitimacy with clinical priorities, and care needs with household resource constraints. The study therefore suggests that rural palliative care should be developed through context-sensitive models that identify locally meaningful sources of trust, care, obligation, and support rather than assuming that institution-based models can be transferred unchanged across settings.

### Limitations

This study has several limitations. First, although participants were recruited from multiple provinces and one municipality, the sample size within each region was small and unevenly distributed. China is geographically, culturally, and economically diverse, and meaningful comparisons across regions, provinces, or ethnic groups were not possible in the present study. The findings should therefore be interpreted as identifying shared patterns within the included rural contexts, rather than as representing all rural communities in China.

Second, the study included both individual interviews and three small group interviews. Although the group interviews were conducted only when individual interviews were not practicable, and the data were reviewed for potential conformity effects, group dynamics may still have influenced how participants discussed sensitive topics such as death, family responsibility, and palliative care.

Third, because palliative care remains unfamiliar to many rural residents, interviewers provided a brief explanation of the concept before the interviews. This step was necessary to support informed discussion, but it may also have shaped participants’ subsequent responses.

Finally, because data were collected concurrently across multiple sites, saturation was assessed during analysis rather than used prospectively to determine the endpoint of recruitment. Future studies could use more iterative data collection and analysis, and could examine regional, ethnic, and socio-economic variation in rural understandings of palliative care.

## Conclusions

This study developed the “Rural Belief about Good Death” framework to explain how rural Chinese residents understand and evaluate palliative care through familism, attachment to ancestral homeland, traditional filial piety, and family economic security. The framework shows that attitudes towards palliative care are shaped not by medical considerations alone, but by culturally, morally, relationally, and economically situated understandings of a good death. Rural palliative care in China should therefore move beyond the simple transplantation of externally developed models towards locally responsive service development. This requires home-oriented care, culturally meaningful public education, accessible professional support, and government-backed financial protection that respond to local understandings of family, place, filial responsibility, and economic security. By integrating these dimensions, the framework provides a culturally grounded basis for developing more responsive and accessible palliative care in rural China, while offering broader insights for rural and resource-limited settings where care preferences are similarly shaped by the interaction of local values, family responsibilities, and material constraints.

## Supplementary Information

Supplementary Material 1.

Supplementary Material 2.

## Data Availability

The data generated in this study are available from the corresponding author upon reasonable request.
