# Supplementary material for: Understanding rural Chinese residents’ perceptions of palliative care: a grounded theory study of beliefs about a good death
Source: BMC Palliat Care. 2026 Aug 7;25:274. doi: 10.1186/s12904-026-02275-x (PMC13584263; doi:10.1186/s12904-026-02275-x)
Supplement: Supplementary file 1 [file 12904_2026_2275_MOESM1_ESM.docx]

**Interview Guides**

1. Have any elderly persons passed away in the village in the past two years? Could you tell us about these individuals? (Cause of death, illness, whether hospitalized, costs incurred, the condition of the patient at the time of death, etc.)
2. People often envy those elderly who are considered “blessed”. What do you think constitutes a “blessed” life for an elderly person?
3. For an elderly person with late-stage cancer and no hope of recovery, when their condition deteriorates, do you think they should be sent to the hospital for rescue treatment? Why?
4. If a doctor suggests inserting tubes or performing surgery on a late-stage cancer patient to help prolong life, do you think their family would agree? Why?
5. Are there any elderly persons in our village who are bedridden long-term due to illness and cannot care for themselves? (If so, what is their living condition like?)
6. What do you think would be the best possible outcome for those bedridden elderly in the village?
7. Elderly persons with incurable diseases often suffer greatly at the end. If nearby hospitals (e.g., township health centers, county hospitals) could provide palliative care services—which, while not life-saving, could alleviate the patient's suffering, albeit at some cost—do you think their families would be willing to send the elderly for such care? Why?
8. For cancer patients, what do you think are their main concerns or worries? And how do you think we can help them cope with these issues?
9. If you were in such a situation, would you want your family to send you for palliative care? Why?
10. In caring for terminally ill patients in rural areas, what aspects do you think are done well? Why?
11. In caring for terminally ill patients in rural areas, what difficulties exist? What aspects are unsatisfactory?
12. Could you briefly share your views on palliative care services?
