# Supplementary material for: Understanding rural Chinese residents’ perceptions of palliative care: a grounded theory study of beliefs about a good death
Source: BMC Palliat Care. 2026 Aug 7;25:274. doi: 10.1186/s12904-026-02275-x (PMC13584263; doi:10.1186/s12904-026-02275-x)
Supplement: Supplementary file 2 [file 12904_2026_2275_MOESM2_ESM.docx]

**Codebook for Axial and Open Coding**

| **Axial Codes**  **(Reference Points)** | **Open Codes** | **Text Examples (Data sources)** | **Reference Points** |
| --- | --- | --- | --- |
| **Views on Life and Death (89)** | 1) Cherishing life and fearing death | *The older you get, the more you fear death, the more you want to live. (C15)* | 32 |
|  | 2) Falling leaves returning to the root | *For folks in rural China, they still hold the belief that they ought to be buried in their ancestral hometown when they pass away. (C17)* | 20 |
|  | 3) Life and death as natural | *Well, that’s life - everyone goes through birth, old age, sickness and death. It’s just the natural order of things; you can’t fight it; it’s bound to happen. (C11)* | 17 |
|  | 4) Fearlessness towards death | *I’m not afraid. Everyone has to walk this path eventually. Everyone is on the way to death, there’s no young or old. (C16)* | 8 |
|  | 5) Dying peacefully in old age | *When your time’s up, you pass away just like falling asleep, without any pain whatsoever. (C11)* | 5 |
|  | 6) One’s fate is not in one’s own hands | *You can’t call the shots on the fate of your life, and no one can ever really figure out what fate’s all about. (C32)* | 4 |
|  | 7) Death as liberation | *Once dead, all worries are gone. (C23)* | 3 |
| **Awareness of Quality of Life and Dignity (118)** | 1) Family Fulfillment | *Family harmony is what brings happiness. (C2)* | 39 |
|  | 2) Physical and mental well-being | *Good health is the real foundation of a happy and comfortable life. (C37)* | 35 |
|  | 3) Dying without pain | *When it really comes to an end, what matters is to pass away painlessly and with dignity. For an elderly person, that’s the greatest fortune and the deepest desire in their hearts. (C17)* | 14 |
|  | 4) No lack of material resources | *Of course, material things are indispensable. If you can’t even put food on the table or fill your stomach, you certainly can’t talk about happiness. (C18)* | 12 |
|  | 5) Avoiding futile treatment | *When it truly comes to the final moments of life, they certainly don’t want to lose their dignity - being attached to tubes all over, spending all their money on the hospital for nothing, and still having to leave in the end. (C17)* | 9 |
|  | 6) Longevity | *We say anyone who lives a long life is blessed. (C11)* | 5 |
|  | 7) Not being a burden to family and children | *Truth be told, not being a burden to your children is the true blessing. (C17)* | 4 |
| **Perceptions of Medical Care (85)** | 1) Patient care relying on the family | *I believe the biggest strength of rural people is their strong family bonds, and family members take excellent care of one another. (C37)* | 55 |
|  | 2) Treating to the end to fulfill filial piety | *In rural areas now, rural families won’t easily give up on treatment. Because living conditions have improved compared to the past, their children are financially capable now and also very filial. Even when a patient is in the advanced stage of illness and doctors say treatment is no longer effective, the children will still insist on continuing it. (C6)* | 19 |
|  | 3) Patient care through neighborhood mutual assistance | *When the children get tired from their caregiving sometimes, you can ask the neighbors to come over to accompany the patient and chat a bit together. (C13)* | 8 |
|  | 4) Prioritizing treatment for the young over the elderly | *Nowadays, most people spare no effort to treat sick kids no matter what. Their attitude toward the elderly is different, though. Fewer spare no effort to treat them. This is human instinct. (C32)* | 3 |
| **Customs for Handling Postmortem Matters (20)** | 1) Dying at home | *It’s almost always at their own home for rural families - passing away outside is considered bad luck. (C14)* | 8 |
|  | 2) Family members staying by the dying person’s side | *In our rural areas, we must stay by their side, no matter when they pass, someone must be there with them. (C13)* | 4 |
|  | 3) Being laid to rest in peace | *The deceased finds peace only when interred in the earth. (C22)* | 3 |
|  | 4) Purchasing burial items and dressing the deceased in burial clothes | *Once he passed away, we helped to buy those things, we went to town to buy the mourning clothes, all the clothes for the dead, and the joss paper. (C13)* | 3 |
|  | 5) Not disturbing the deceased | *Don’t move them when they are dying; they are already in great pain. The soul is separating from the body. If you cry and move them then, they suffer more. Right after death, you shouldn't cry; if you cry, they can’t let go and won’t depart peacefully. (C15)* | 2 |
| **Attitudes Toward Palliative Care (64)** | 1) Positive acceptance of palliative care | *This matter of palliative care, now that the state is promoting it, I think it’s a good thing and should be strongly supported. (C6)* | 47 |
|  | 2) Rejection of palliative care | *Not very useful, a superficial project, just do some practical action. (C2)* | 17 |
| **Influence of Economic Conditions on Acceptance of Palliative Care (23)** | 1) Poverty hindering rural residents from accepting palliative care | *Rural folks have no money in hand. The problem is having no money. (C33)* | 13 |
|  | 2) Accepting palliative care only if costs are low | *A small amount of money is acceptable, but none of us are willing or able to afford a large sum, right? (C32)* | 10 |
| **Needs for Palliative Care Services (40)** | 1) Hoping for professional service support | *I think because it’s in rural areas, nearby palliative care facilities are relatively few. Places that actually implement palliative care are almost non-existent. (C20)* | 35 |
|  | 2) Expecting home-based care | *I think palliative care in a hospital is not as convenient as at home. (C35)* | 5 |
| **Expectations for Government Support and Promotion Policies (37)** | 1) Strengthening policy support | *Looking forward to government policies coming through, so there can be professional teams, and we can enjoy this kind of care - this is also an expectation of ours. (C32)* | 17 |
|  | 2) Government increasing financial investment | *Palliative care is good, but it simply won’t work out without government funding. (C4)* | 12 |
|  | 3) Hoping for life-and-death education and palliative care publicity | *I think the government needs to do more to promote education about palliative care, such as playing promotional videos in the villages and holding awareness sessions like they did before, so that everyone can understand what palliative care is all about. (C19)* | 8 |
